# Trophic niches of native and nonnative fishes along a river-reservoir continuum

**DOI:** 10.1038/s41598-021-91730-1

**Published:** 2021-06-09

**Authors:** Casey A. Pennock, Zachary T. Ahrens, Mark C. McKinstry, Phaedra Budy, Keith B. Gido

**Affiliations:** 1 Department of Watershed Sciences and The Ecology Center, Utah State University, Logan, UT 84322 USA; 2 Division of Biology, Kansas State University, Manhattan, KS 66502 USA; 3 Utah Division of Wildlife Resources, Moab, UT 84532 USA; 4 Upper Colorado Regional Office, U.S. Bureau of Reclamation, Salt Lake City, UT 84138 USA; 5 U.S. Geological Survey, Utah Cooperative Fish and Wildlife Research Unit, Utah State University, Logan, UT 84322-5290 USA

**Keywords:** Stable isotope analysis, Food webs, Freshwater ecology

## Abstract

Instream barriers can constrain dispersal of nonnative fishes, creating opportunities to test their impact on native communities above and below these barriers. Deposition of sediments in a river inflow to Lake Powell, USA resulted in creation of a large waterfall prohibiting upstream movement of fishes from the reservoir allowing us to evaluate the trophic niche of fishes above and below this barrier. We expected niche overlap among native and nonnative species would increase in local assemblages downstream of the barrier where nonnative fish diversity and abundance were higher. Fishes upstream of the barrier had more distinct isotopic niches and species exhibited a wider range in δ^15^N relative to downstream. In the reservoir, species were more constrained in δ^15^N and differed more in δ^13^C, representing a shorter, wider food web. Differences in energetic pathways and resource availability among habitats likely contributed to differences in isotopic niches. Endangered Razorback Sucker (*Xyrauchen texanus*) aggregate at some reservoir inflows in the Colorado River basin, and this is where we found the highest niche overlap among species. Whether isotopic niche overlap among adult native and nonnative species has negative consequences is unclear, because data on resource availability and use are lacking; however, these observations do indicate the potential for competition. Still, the impacts of diet overlap among trophic generalists, such as Razorback Sucker, are likely low, particularly in habitats with diverse and abundant food bases such as river-reservoir inflows.

## Introduction

Nonnative species have been widely introduced into freshwater habitats, presenting one of the most significant and persistent threats to native species^1–3^. Nonnative fishes, for example, can have wide ranging effects^4^, including altering energetic pathways and changing food web structure^5,6^. Gauging the impact of biological invasions is relatively straightforward for top predators that consume native species^7^, but assessing impacts occurring through indirect effects of competition are more difficult to identify. Evidence from stable isotope analysis suggests that native species often shift their trophic niche in the presence of nonnative species, presumably to less preferred, lower quality resources^8–10^. Competitive interactions between species with similar trophic niches occupying the same habitat might lead to exclusion unless species partition resources^11–13^, but direct evidence of competition in the wild is difficult to detect^14,15^. Still, food web studies can provide important information on potential species interactions and elucidate impacts of nonnative species on native assemblages^10,15^.

Fish assemblage and food web structure can change across habitat and trophic resource gradients that shift longitudinally in rivers^16–18^, but these gradients are often altered by river regulation^19,20^. Construction of dams and reservoirs have transformed riverscapes into a mosaic of lotic and lentic habitats^21^, frequently leading to changes in fish assemblage composition both downstream and upstream of dams^22^. Further, water development has resulted in discontinuities in resource gradients^23–25^, created new resource gradients^21,26^, and facilitated introduction and proliferation of nonnative species^27^. These alterations have changed natural habitat gradients (e.g., habitat templates^28^) and increased the number of potential competitors^29–31^. Habitat alterations have also led to reduction and homogenization of niche space resulting from decreased trophic diversity and shortening of food chains in some river systems^32^, while species introductions have caused lengthening of food chains in others^9,33,34^.

Our understanding of trophic resource use by some imperiled fishes and their overlap with nonnative fishes is limited. Predation and competition are the most often cited effects of nonnative species^4,35,36^, yet evidence of competition is often missing in research of freshwater systems, partially because of a lack of data on trophic resource use and availability. Moreover, because of their rarity and conservation status, it is often difficult to directly assess the diet of imperiled species^37–39^, limiting our understanding of impacts of nonnative species as resource competitors. Non-lethal and less intrusive techniques, such as stable isotope analysis, provide an alternative approach to compare overlap in trophic resource use of fishes^9,10,40^. For instance, δ^13^C reflects organic matter sources, such as autochthonous versus allochthonous primary production in streams^41^ or pelagic versus benthic primary production in lakes^42^, but the δ^13^C values of these sources can sometimes overlap^43^. Meanwhile, δ^15^N can provide an estimate of trophic position^44,45^, and several informative analytical metrics have been developed to quantify isotopic niche breadth (realized niche) and trophic overlap among species in an assemblage^46–48^.

Nonnative species introductions and habitat alteration are the leading hypothesized drivers for native fish imperilment in the Colorado River basin in the American Southwest^35,49^. Novel habitats in the basin, such as large water-storage reservoirs, serve as sources for nonnative species, but some native species also appear to be using river-reservoir inflow habitats^50,51^. Previous research identified overlap in resource use between younger life stages of native (e.g., Flannelmouth Sucker *Catostomus latipinnis*) and nonnative species (e.g., Common Carp *Cyprinus carpio*) in the upper Colorado basin^9,52,53^, but data on large-river, federally endangered species, such as Colorado Pikeminnow (*Ptychocheilus lucius*) and Razorback Sucker (*Xyrauchen texanus*) are more limited, likely because of their rarity in the system.

We characterized isotopic niches of species across a river-reservoir continuum in habitats differing in relative abundance of native and nonnative species. Deposition of sediments from the San Juan River at the inflow to Lake Powell on the Colorado River, USA and new channel formation following declines in reservoir water level resulted in the creation of a large waterfall that prohibits the upstream migration of fishes from the reservoir^54,55^. This created a rare opportunity to compare relative positions of species in trophic niche space in the river above this barrier, where the assemblage is dominated by native fishes, and in the river and reservoir below the barrier, where the assemblage is dominated by nonnative fishes. We had three objectives in this study. First, we identified differences in mean isotopic signatures of fishes within habitats, and expected species isotopic niches would reflect differences in resource use. Second, we quantified isotopic niche breadth of species within habitats, and predicted wider isotopic niche breadth for all species in the reservoir, because this habitat consists of areas with both detritus-based and algae-based food webs (e.g., reservoirs consist of lotic and lentic habitats^26^). Finally, we assessed niche overlap between pairs of native and nonnative fishes within habitats. We predicted trophic niche overlap between native and nonnative species would be greatest in the reservoir, because of the higher diversity and relative abundance of nonnative fish species (i.e., more potential competitors), and predicted overlap would decrease moving upstream into riverine habitats. We were primarily interested in assessing isotopic niche overlap among *X. texanus* and other benthic omnivores that might occupy a similar trophic position (e.g., fish from the same trophic guild are functionally similar^56^). We also included data on *P. lucius* because of its conservation status, and a lack of data on trophic resource use of this species in the wild^53^. Both species are the focus of conservation and recovery efforts and use riverine and reservoir inflow habitats in the basin^50^. Tissues from other nonnative species (fishes and quagga mussels *Dreissena rostriformis bugensis*) were collected, but in only one of the three habitats so we excluded these from analyses and present these data for reference in the Electronic Supplementary Material (Supplementary Table S1, Fig. S1).

## Methods

### Study area

**Figure 1:**
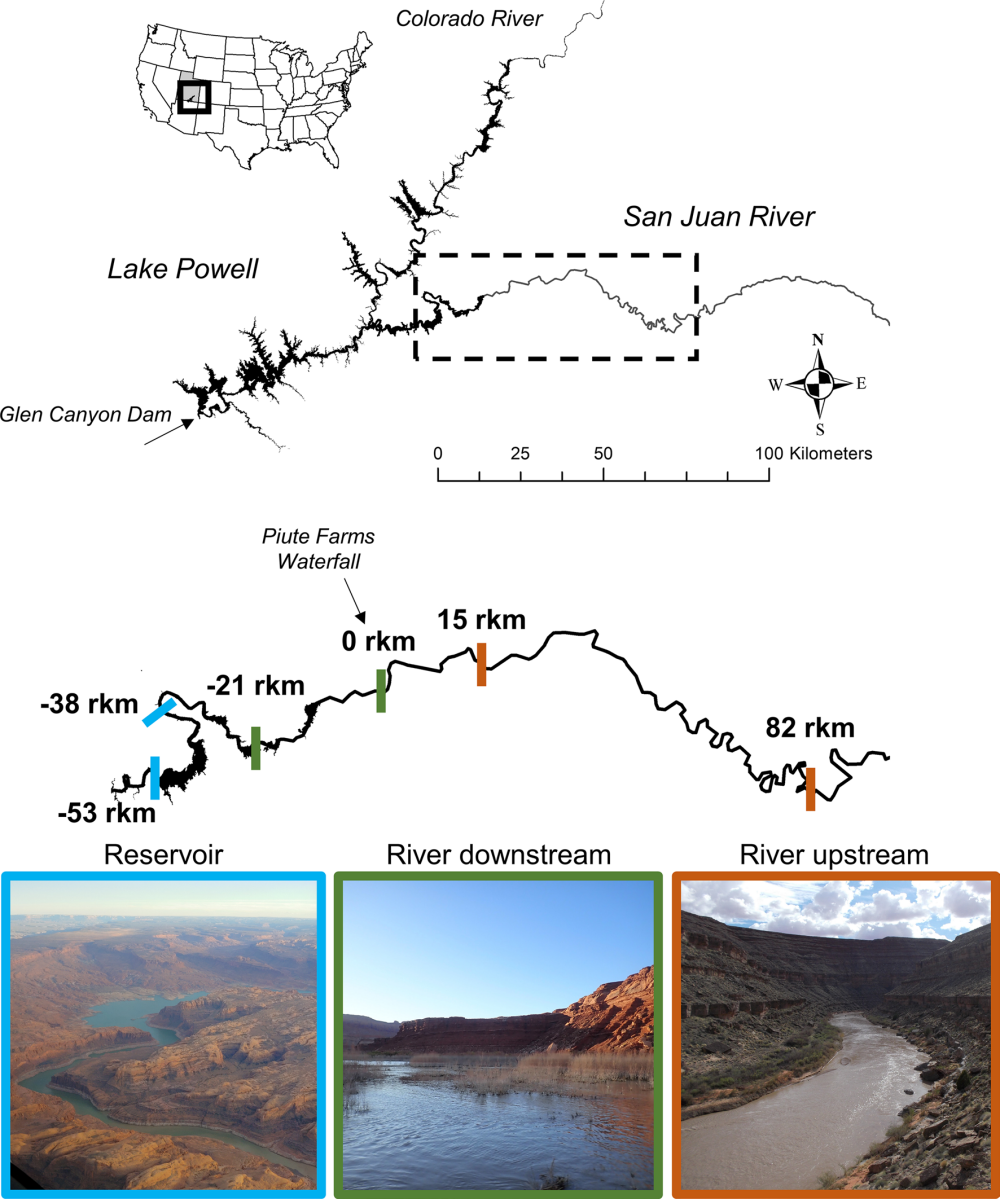
Fish tissues for isotope analyses were collected over 135 river kilometers (rkm) from three habitats along the lower San Juan River and into Lake Powell in the Colorado River Basin, southeastern Utah, USA. Habitats were delineated in relation to the Piute Farms Waterfall (0 rkm) on the San Juan River and to the San Juan River inflow to Lake Powell. Colored bars represent the extent of sampling in each habitat. Reservoir habitat photograph by Douglas Turnquist, used with permission. River downstream and river upstream photograph by Casey Pennock.

In 2018 and 2019, we sampled fishes and collected tissues for stable isotope analysis across 135 river kilometers (rkm) of the San Juan River, including the San Juan River arm of Lake Powell in southeastern Utah (Fig. 1). The San Juan River begins in the Rocky Mountains in southwestern Colorado and flows south and west across parts of Colorado, New Mexico, and Utah before its eventual confluence with the Colorado River, which is now inundated by Lake Powell. The historical confluence is approximately 30 rkm downstream of the lower extent of our study area. We sampled three habitats along the river-reservoir continuum (Fig. 1). The upstream habitat (hereafter river upstream; 67 rkm) occurred from Mexican Hat, Utah to approximately 15 rkm upstream of the Piute Farms Waterfall, a 6 m tall geomorphic barrier to upstream fish movement that has emerged on the San Juan River as a result of sediment deposition during higher water levels in Lake Powell, and subsequent superimposition processes through delta sediments^55^. This habitat was mainly canyon-bound and higher gradient, lotic habitat (mean gradient = 1.6 m km^−1^, measured from Google Earth). The middle habitat began directly below the Piute Farms Waterfall (hereafter river downstream) and included 21 rkm of the San Juan River and Lake Powell inflow. This habitat consisted of lower gradient (0.6 m km^−1^), slowing velocity, and turbid lotic habitats at the upper end of the reservoir (the riverine zone; *sensu*^26^). The last, and most downstream, habitat also occurred in the San Juan River–Lake Powell inflow across 15 rkm, and encompassed gradients of decreasing turbidity and increasing channel width and depth (here after reservoir)^51^.

### Stable isotope tissue collection

We collected pectoral fin tissues from the distal end of the fin for isotope analysis from fishes captured in the river upstream and river downstream habitats in March (13–31), April (1–2), June (8–10), and September (22–28) in 2018 using raft electrofishing (ETS Electrofishing Systems, Madison, Wisconsin), and from reservoir fishes in April and May 2019 captured in trammel nets (45.2 m long × 1.2 m tall with 25 mm internal bar mesh and 305 mm outer bar mesh). Fin tissues typically have an isotopic half-life of several months for both δ^13^C and δ^15^N ^57–59^. In the river, we sampled fish in 0.1–3.2 km increments depending on fish densities to reduce handling stress on native fishes. We sampled fish in the reservoir by placing a trammel net in every 0.5 km segment of shoreline along approximately 20 km of the historic river channel^51^. The US Fish and Wildlife Service stocks PIT-tagged *X. texanus* every fall in the San Juan River, and therefore we removed individuals stocked the previous fall from analyses to avoid influence of residual hatchery diets on isotope signatures. All sampling was performed in accordance with the relevant guidelines and regulations, reviewed and approved by the Utah State University Institutional Animal Care and Use Committee Animal Care and Ethics Committee (#10,169), and permitting from the U.S. Fish and Wildlife Service (TE067729-6), Navajo Nation (Special Permit, Scientific Collecting 1187), and National Park Service (GLCA-2017-SCI-0003).

We either air-dried tissues or preserved them in table salt in the field. Following^60^, we prepared salt-preserved tissues by rinsing tissues with distilled water and soaking them in distilled water for 4 h. In the laboratory, we dried tissues (60 °C for ~ 8 h), homogenized tissues with a mortar and pestle, and packed samples into tin capsules, and weighed to the nearest 0.1 mg. We analyzed tissues for carbon and nitrogen stable isotope ratios. Tissues collected from the reservoir were analyzed at the Stable Isotope Mass Spectrometry Laboratory, Kansas State University, Manhattan, Kansas by continuous-flow, direct combustion, and mass spectrometry using an Elementar Vario Cube elemental analyzer coupled to an Elementar Vision mass spectrometer (Elementar Americas, Mt. Laurel, New Jersey). Tissues collected from the river upstream and river downstream habitats were analyzed at the Utah State University Department of Geology Stable Isotope Laboratory, using a Thermo Scientific Gasbench II or Costech ECS4010 elemental analyzer (Costech Analytical Technologies Inc., Valencia, California) coupled to a Thermo Scientific Delta V Advantage IRMS (Thermo Fisher Scientific, Walmath, Virginia). We report data on a per mille basis (‰) in delta (δ) notation. We calculated delta values as:

$$\updelta ^{13} {\text{C or}}\updelta ^{15} {\text{N}} = \left[ {(R_{sample} /R_{standard} ) - 1} \right] \times 1,000$$

where *R* is equal to ^13^C/^12^C and ^15^N/^14^N. We used laboratory standards calibrated against international standards: Pee Dee Belemnite was the standard for carbon and atmospheric molecular nitrogen was the standard for nitrogen. Measurement error on routine analysis of laboratory standards was less than 0.50‰ for both δ^13^C and δ^15^N. For comparison, we also ran duplicate samples (*n* = 4) at both stable isotope laboratories and these differed on average by 1.4‰ (CV = 0.4) for δ^13^C and 0.58‰ (CV = 0.08) for δ^15^N.

### Statistical analysis

#### Fish assemblage variation among habitats

To assess fish assemblage composition across our study area, we quantified variation in relative abundance of native species across our three habitats. We used species relative abundance because sampling methods differed between the river (raft electrofishing) and reservoir (trammel netting) habitats. We first transformed catch data into catch per unit effort (fish h^−1^), and then calculated relative abundance as the catch per unit effort of native species divided by the total catch per unit effort. Both capture techniques tend to target large-bodied fishes and are biased against smaller fishes^61^; therefore, we removed Red Shiner (*Cyprinella lutrensis*) and Western Mosquitofish (*Gambusia affinis*), two small-bodied species, from the dataset before calculating relative abundance. We tested for differences in native species relative abundance among habitats using a linear mixed effect model using the lme4 package^62^. We logit transformed proportional data prior to fitting the model, and included reach as a fixed factor and a random intercept of month nested within year. We used likelihood ratio tests with the car package^63^ to estimate statistical significance (*α* = 0.05).

#### Within reach isotopic variation

We tested for differences in species isotopic niche positions within habitats by separately comparing values of δ^13^C and δ^15^N of each species using linear mixed effects models. Statistical significance was assessed using likelihood ratio tests as described above. We used linear mixed effects models and included species as a fixed factor and fish length as a random effect. There was some variation among the seasonal representation of some species in the river upstream and river downstream habitats (Supplementary Table S2), but the majority of fishes were present across seasons. To account for temporal variation, we included a random effect of month of sample collection. However, the random effect of month had a variance of zero in the reservoir models and the random effect of fish length had a variance of zero in the river downstream models and the δ^13^C model in the river upstream. We used residual plots to check adherence to model assumptions and found no clear violations of normality or heteroskedasticity. We used Tukey’s HSD to make pairwise comparisons between species following a significant global model (*α* = 0.05). Finally, using the piecewiseSEM package^64^, we calculated variance explained by fixed factors using marginal *R*^2^ and fixed and random factors together using conditional *R*^2^^65^.

#### Within reach isotopic niche breadth and overlap

We used standard ellipse area of δ^13^C and δ^15^N as a measure of isotopic niche breadth of each species in each reach. We calculated standard ellipse areas corrected for sample sizes (SEAc) using the SIBER package^47^. Standard ellipses represent the core trophic niche of a species and encompass roughly 40% of the samples in a group regardless of the sample size^47^. To estimate niche overlap between native and nonnative species pairs in each reach we used the nicheROVER package^66^. This analysis uses a Monte Carlo resampling routine (n = 10,000 draws) to randomly draw from the sampled population and calculates a mean and 95% credible interval of niche overlap probabilities. These values are directional in that they represent the estimated probability a randomly drawn individual of one species overlaps with the niche of another species in δ^13^C and δ^15^N bivariate space^48^. All analyses were conducted using the R statistical language version 3.5.3^67^.

## Results

**Figure 2:**
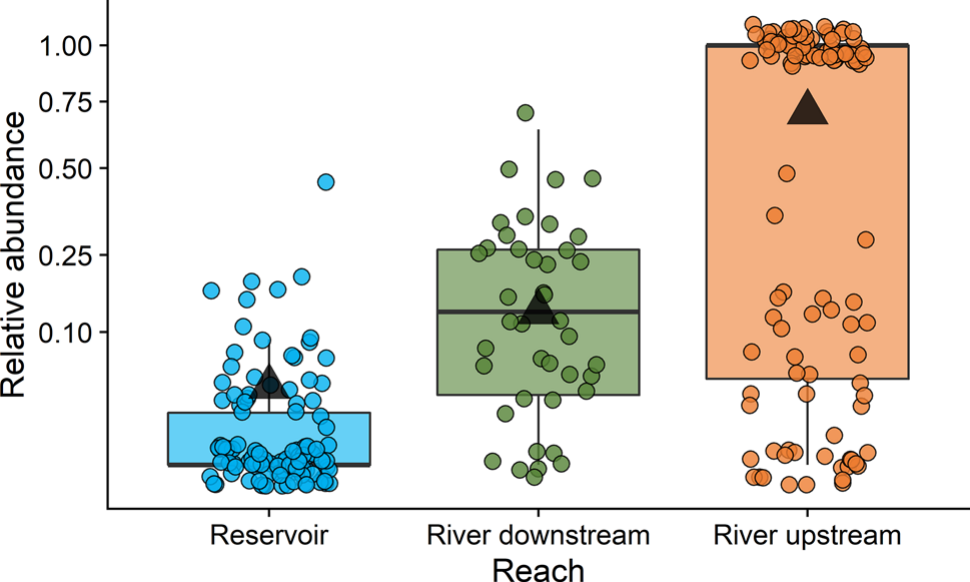
Relative abundance of native species in fish assemblages across the San Juan River–Lake Powell continuum from the reservoir, to the river downstream, to the river upstream. Triangles represent marginal means from a linear mixed effects model including reach as a fixed factor and a random effect of month nested within year, the bold lines are medians, box edges are the 25th and 75th quartiles, and whiskers extend from box edges to the smallest and largest value no further than 1.5 × the interquartile range. Points represent 0.1–3.2 km river reaches sampled or individual trammel nets in the reservoir. A small amount of jitter was added to points to reduce overlap. The y-axis has been scaled with a square root transformation to better show small proportions.

Fish assemblage composition changed distinctly along our study area. As expected, nonnative species were more dominant towards the reservoir and relative abundance of native species declined from upstream to downstream (*F*_2,112.87_ = 21.37, *P* < 0.001; Fig. 2). Six nonnative species were only captured downstream of the waterfall in the river downstream and reservoir habitats, and most nonnative fish were in higher abundance downstream of the waterfall (Supplementary Fig. S1). For example, average catch per unit effort of *C. carpio* was 4× greater in the river downstream than the river upstream. The one exception was Channel Catfish (*Ictalurus punctatus*), which was 20× more abundant upstream of the waterfall relative to the river downstream in late March and early April.

**Figure 3:**
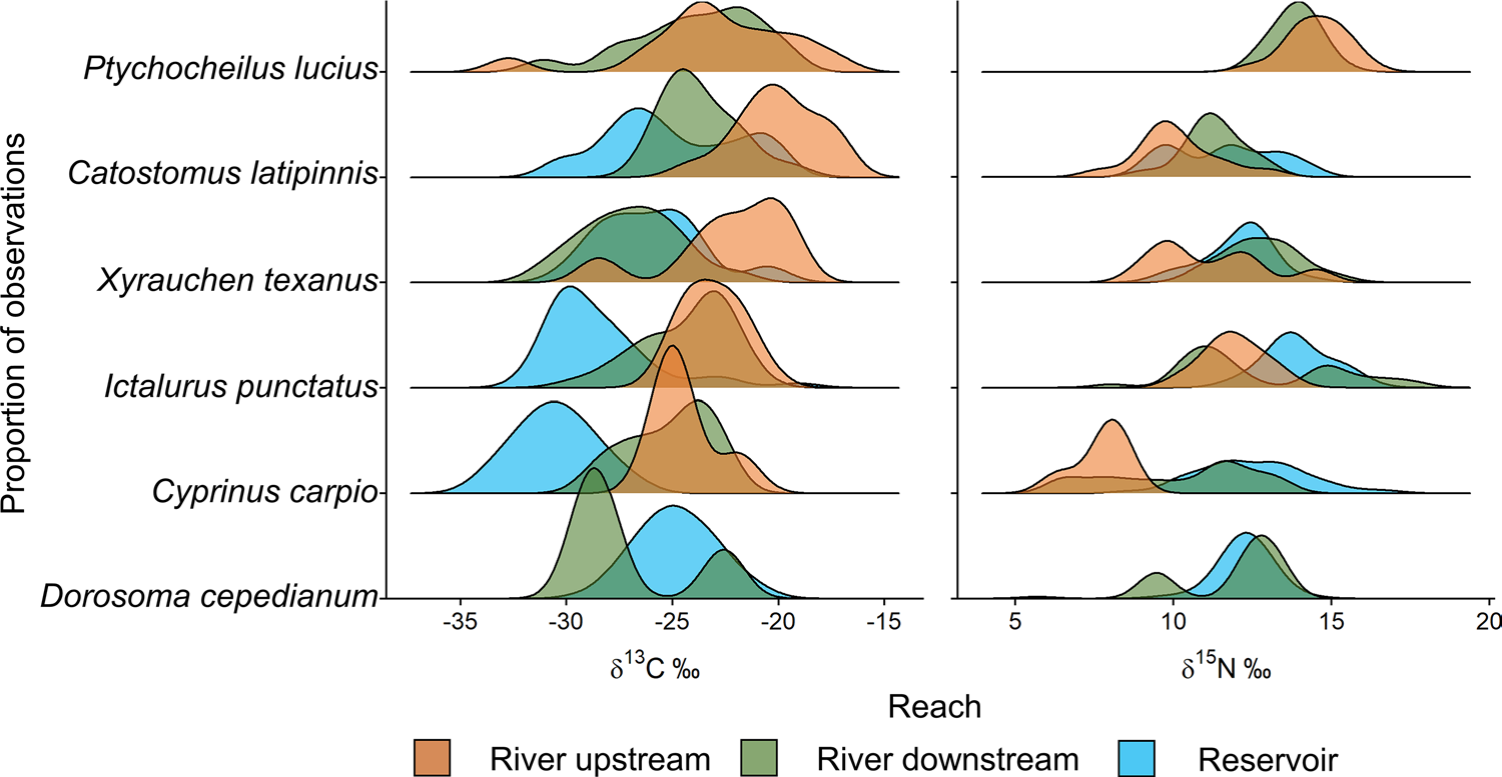
Density ridgeline plots showing distribution of δ^13^C and δ^15^N of species among the three sampled habitats in the San Juan River and Lake Powell reservoir. The top three species are native to the Colorado River basin.

Isotopic signatures indicated differences in trophic resource overlap across the river-reservoir continuum (Fig. 3). We collected tissues from 68 fishes in the river upstream, 108 in the river downstream, and 186 in the reservoir (Table 1). Means of δ^13^C were statistically different among some species within the river downstream and reservoir habitats (Table 2), but there were no statistical differences among species in the river upstream (*P* = 0.094). δ^15^N values, on average, differed significantly among some species in all habitats (Table 2). The fixed effect of species explained relatively little variation in δ^13^C in the river upstream and river downstream (marginal *R*^2^ = 0.09–0.16) compared to the reservoir (marginal *R*^2^ = 0.54). Conversely, the fixed effect of species contributed to a relatively low proportion of the explained variation in δ^15^N in the reservoir (marginal *R*^2^ = 0.26) compared to the river upstream (marginal *R*^2^ = 0.69) and river downstream (marginal *R*^2^ = 0.31; Table 2). In the reservoir, species were more constrained in δ^15^N, and only *I. punctatus* had a significantly different δ^15^N signature from other species based on *post-hoc* tests (Table 1). In sum, relative differences among species in the river upstream were driven more by differences in δ^15^N; in the reservoir differences were more pronounced in δ^13^C. Differences among species in the river downstream appeared somewhat intermediate between the river upstream and the reservoir (Fig. 3).

**Table 1:** Marginal mean estimates of δ^13^C and δ^15^N from linear mixed effects models, standard ellipse area corrected for sample size (SEAc), fish size (mm total length), and number of fish analyzed (*n*) from fishes captured in the San Juan River and Lake Powell, Utah.

Habitat/Species	δ^13^C (mean, SE)	δ^15^N (mean, SE)	Total length (mean ± SD)	SEAc	*n*
**River upstream**					
Channel Catfish (*Ictalurus punctatus*)	− 23.3 (1.0)^a^	11.8 (0.4)^a^	304 ± 123	3.69	27
Colorado Pikeminnow (*Ptychocheilus lucius*)*	− 22.5 (1.1)^a^	14.4 (0.4)^b^	295 ± 74	11.36	14
Common Carp (*Cyprinus carpio*)	− 23.9 (1.4)^a^	7.9 (0.6)^c^	567 ± 64	2.24	5
Flannelmouth Sucker (*Catostomus latipinnis*)*	− 21.7 (1.2)^a^	10.4 (0.5)^d^	251 ± 161	8.68	14
Razorback Sucker (*Xyrauchen texanus*)*	− 21.3 (1.3)^a^	11.3 (0.5)^ad^	408 ± 83	7.96	8
**River downstream**					
Channel Catfish (*I. punctatus*)	− 24.1 (0.9)^a^	12.7 (0.5)^ac^	251 ± 177	10.62	32
Colorado Pikeminnow (*P. lucius*)	− 23.7 (1.0)^a^	13.8 (0.6)^a^	413 ± 95	5.11	15
Common Carp (*C. carpio*)	− 24.4 (1.0)^a^	10.0 (0.6)^b^	422 ± 217	13.77	22
Flannelmouth Sucker (*C. latipinnis*)*	− 23.1 (1.0)^a^	11.2 (0.6)^bc^	365 ± 76	5.40	16
Gizzard Shad (*Dorosoma cepedianum*)	− 26.1 (1.3)^ab^	11.5 (1.0)^abc^	354 ± 161	4.29	4
Razorback Sucker (*X. texanus*)*	− 26.4 (1.0)^b^	12.7 (0.6)^ac^	511 ± 53	6.46	19
**Reservoir**					
Channel Catfish (*I. punctatus*)	− 28.5 (0.3)^a^	14.0 (0.2)^a^	320 ± 74	8.47	45
Common Carp (*C. carpio*)	− 30.5 (0.3)^b^	12.5 (0.2)^b^	489 ± 51	9.52	47
Flannelmouth Sucker (*C. latipinnis*)*	− 25.0 (0.7)^c^	11.6 (0.4)^b^	417 ± 54	12.94	11
Gizzard Shad (*Dorosoma cepedianum*)	− 24.8 (0.3)^c^	12.1 (0.2)^b^	450 ± 48	6.69	59
Razorback Sucker (*X. texanus*)*	− 26.0 (0.4)^c^	12.1 (0.3)^b^	481 ± 60	7.39	24

Native species are denoted with an*. Within habitats, species sharing letters did not differ (Tukey’s HSD: *P* > 0.05) in mean isotope values.

**Table 2:** Test statistics from linear mixed effects models testing for differences in δ^13^C and δ^15^N among species within habitats.

Habitat/isotope	Likelihood ratio test statistics			Marginal *R*^2^	Conditional *R*^2^
	*F*	*df*	*P*		
**River upstream**					
δ^13^C	2.09	4, 58.8	0.094	0.09	0.41
δ^15^N	33.93	4, 50.8	< 0.001	0.69	0.79
**River downstream**					
δ^13^C	5.81	5, 96.12	< 0.001	0.16	0.44
δ^15^N	9.77	5, 96.39	< 0.001	0.31	0.41
**Reservoir**					
δ^13^C	50.13	4, 135.26	< 0.001	0.54	0.61
δ^15^N	15.54	4, 122.04	< 0.001	0.26	0.28

Mixed effects models included a random effect of month.

**Figure 4:**
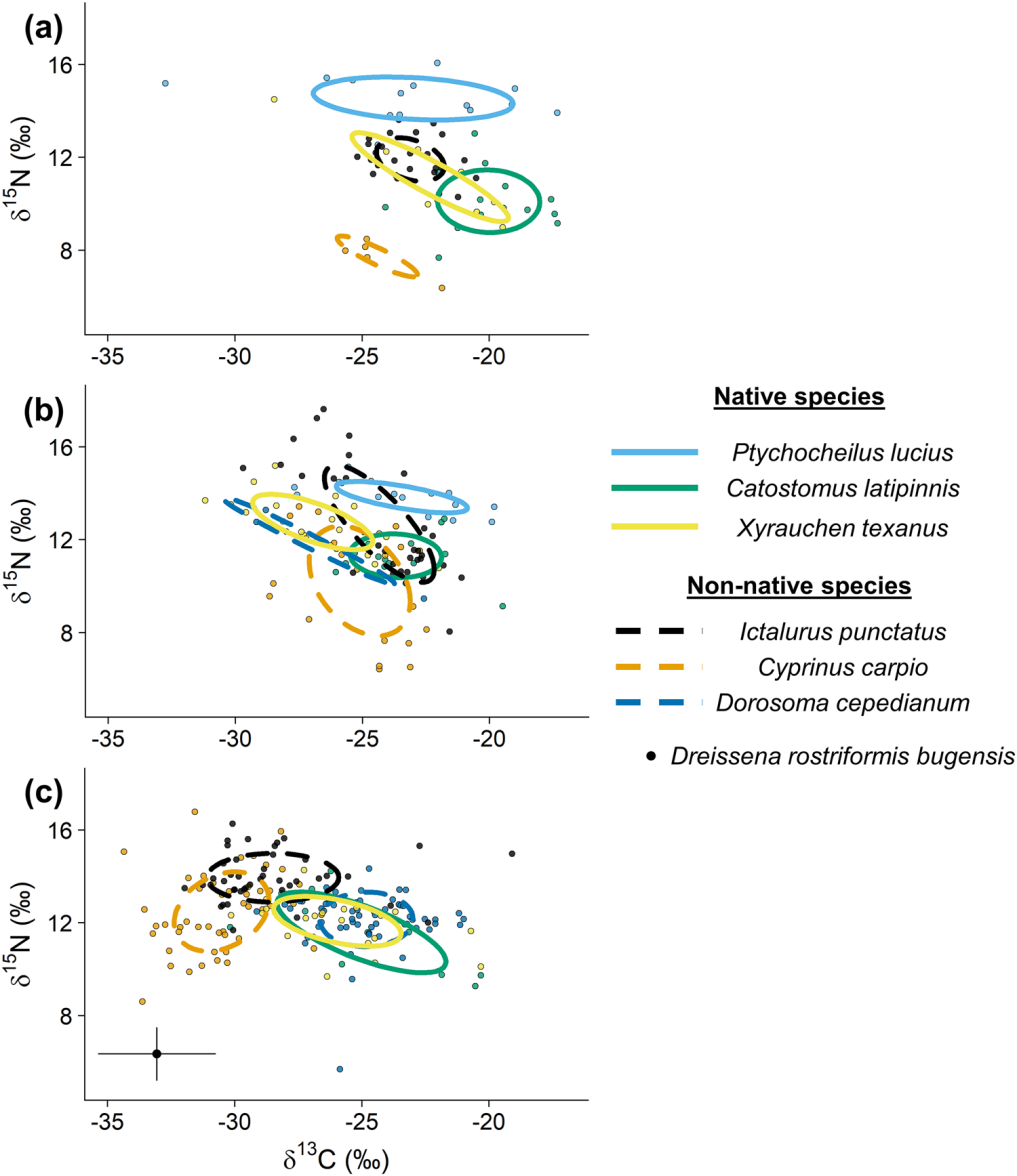
Isotope biplots from fish assemblages in the river upstream on the San Juan River, Utah (**a**), river downstream (**b**), and in Lake Powell reservoir (**c**). Ellipses are standard area ellipses corrected for samples size (SEAc; Jackson et al. 2011). Some species present in assemblages differ among panels (i.e., *P. lucius* and *D. cepedianum*). Filter-feeding Quagga mussels (*Dreissena rostriformis bugensis*) were only collected in the reservoir (dot represents the mean and lines are SE).

Isotopic niche breadth generally increased from the river upstream to the river downstream and the reservoir, somewhat matching our prediction that niche breadth would be wider in the Lake Powell inflow. Isotopic niche breadth for native and nonnative species varied over the three habitats (Table 1), and nonnative species showed relatively larger shifts compared to native species (Fig. 4). In the river upstream, both nonnative species exhibited relatively small isotopic niche breadths (2.24 and 3.69) based on SEAc, and the SEAc of native species (mean across species = 9.33) was 3× larger than nonnative species (Table 1). This pattern was not apparent in the river downstream of the waterfall, where SEAc of native species declined relative to the river upstream by 18% for *X. texanus,* 38% for *C. latipinnis*, and 55% for *P. lucius*. Conversely, SEAc of *I. punctatus* increased by 188% and *C. carpio* by 514% relative to the river upstream (Table 1). In the reservoir, SEAc of native *C. latipinnis* was 49% larger relative to the river upstream. The niche breadth of nonnative *I. punctatus* (130%) and *C. carpio* (325%) was still larger compared to the river upstream, but SEAc was 20% and 30% lower relative to the river downstream, respectively. The SEAc of Gizzard Shad (*Dorosoma cepedianum*) increased by 56% from the river downstream to the reservoir. Relative to all other species *X. texanus* had the most consistent niche breadth across habitats ranging from 7.96 in the river upstream to 6.46 in the river downstream to 7.39 in the reservoir (Table 1).

We predicted niche overlap would be highest in the reservoir, but similar to niche breadth niche overlap was actually highest in the river downstream. The probability of niche overlap between native fishes and other native and nonnative fishes varied among habitats, corresponding with increases in the number and relative abundances of nonnative species. Probability of niche overlap among species pairs tended to be highest in the river downstream (Table 3). For instance, the probability of *C. latipinnis* overlapping the niche of *C. carpio* and *I. punctatus* in the river upstream was ≤ 25% on average. In the river downstream, the probability of *C. latipinnis* overlapping the niche of those same two species was 88% on average (Table 3). We observed similar patterns for *P. lucius* and *X. texanus*, whereby the probability of overlap with other species was higher in the river downstream relative to the river upstream. In the reservoir, probabilities of overlap between native and nonnative species remained high relative to the river upstream and ranged from 30–68% and 41–83% for *C. latipinnis* and *X. texanus,* respectively. The highest overlap probabilities across all three habitats occurred between *C. latipinnis* and *X. texanus* in the reservoir (93%; Table 3).

**Table 3:** Mean overlap probabilities (95% credible intervals) from posterior distributions of the probability of species in rows overlapping onto the 95% isotopic niche of species in columns for each habitat sampled in the San Juan River and Lake Powell reservoir.

Habitat/Species	Overlap probability (%)					
River upstream	*I. punctatus*	*P. lucius*	*C. carpio*	*C. latipinnis*	*X. texanus*	
*P. lucius*	11 (2–28)	–	< 1	5 (< 1–29)	14 (< 1–39)	
*C. latipinnis*	25 (8–50)	4 (< 1–19)	1 (0–4)	–	57 (32–87)	
*X. texanus*	58 (32–84)	19 (2–49)	< 1	62 (33–90)	–	
River downstream	*I. punctatus*	*P. lucius*	*C. carpio*	*C. latipinnis*	*D. cepedianum*	*X. texanus*
*P. lucius*	67 (46–86)	–	52 (14–84)	15 (< 1–56)	1 (0–5)	31 (8–64)
*C. latipinnis*	88 (69–98)	7 (1–24)	88 (65–99)	–	19 (6–44)	69 (37–94)
*X. texanus*	61 (37–84)	24 (6–53)	82 (58–98)	45 (21–75)	27 (12–58)	–
Reservoir	*I. punctatus*	*C. carpio*	*C. latipinnis*	*D. cepedianum*	*X. texanus*	
*C. latipinnis*	40 (18–65)	30 (11–53)	–	68 (45–87)	75 (50–94)	
*X. texanus*	56 (34–80)	41 (22–63)	93 (77–99)	83 (68–95)	–	

Estimates are based on 10,000 Monte Carlo iterations. Full species names are provided in Table 1.

## Discussion

Variation in species overlap in isotopic space was apparent along the river-reservoir inflow gradient that coincided with changes in fish assemblage composition and likely variation in diversity of energetic pathways moving from lotic to lentic habitats. Along with increases in dominance of nonnative fishes, we observed increased isotopic niche overlap in the river and reservoir downstream of the waterfall compared to the river upstream where we observed lower nonnative fish relative abundance and occurrence. The Piute Farms Waterfall is a complete barrier to upstream fish movement^55^, and this coupled with nonnative fish removal efforts in the San Juan River has reduced abundance of some nonnative species such as *C. carpio*^68^ that likely move upstream from source populations in the reservoir. Compared to the river upstream, higher overlap in isotopic niche space among species in the river downstream of the waterfall and in the reservoir might indicate chances for competition are higher in these anthropogenic-derived habitats. However, this pattern might simply reflect that these habitats have higher diversity of nonnative fishes (i.e., more potential competitors).

We observed changes in the amount of isotopic overlap between species, and particularly, native and nonnative species among the three habitats. Overlap of species in isotopic niche space can be an indication of potential competition for food resources^69–71^. The two native suckers demonstrated the highest probability of overlap with other species across all habitats, likely due to a more generalist feeding strategy. In contrast, *P. lucius*, a presumed piscivore^72^, exhibited a relatively small probability of overlap with other species, but this was dependent on the habitat. Some nonnative species, such as *I. punctatus*, are hypothesized to limit populations of native fish in the Colorado River basin through predation^73,74^. Our results suggest relatively little overlap between *P. lucius*, the species with the highest δ^15^N in the river upstream and *I. punctatus*, and supports other observations of limited piscivory by *I. punctatus* in the San Juan River^74^. Although we observed more overlap among species in the two habitats downstream of the waterfall, we do not expect competition is a strong driver of assemblage dynamics as river-reservoir inflows are not likely resource-limited^26,75,76^. Because native fishes in the Colorado River basin tend to be trophic generalists^77^, they might be less susceptible to competitive exclusion by nonnative species despite overlap in isotopic niche space. Fishes in the Colorado River basin likely evolved to capitalize on resource availability that varied across space and time, including across lotic-lentic habitat gradients such as the Colorado River Delta^78,79^, expansive reaches of river impounded by lava dams^80^, and lentic habitats created by high water events^81^.

We only had data on baseline trophic levels from the reservoir, which limited our ability to isolate the cause of observed shifts in isotopic niches, which could be due to changes in baseline isotopic signatures, diet, or habitat use. The δ^13^C of filter-feeding quagga mussels in the reservoir was more depleted in samples collected farther in-reservoir (Supplementary Fig. S2), which we hypothesize is due to a shift in basal energy pathways with the food web influenced more by terrestrial inputs (e.g., detritus) in lotic habitat and more by autochthonous resources (e.g., phytoplankton) in lentic habitat. Lotic and lentic habitats differ in dominant energetic pathways of basal resources^41,82,83^, and it appears that, while some fishes had δ^13^C values that were similar in the two river habitats but markedly different from the reservoir (e.g., *I. punctatus*; Fig. 3), others had δ^13^C values that were similar in the reservoir and river downstream but generally different from the river upstream (e.g., *X. texanus*). *Catostomus latipinnis* was the only species that appeared to obtain resources from a mix of river and reservoir resources based on the intermediate δ^13^C values in the river downstream of the barrier. Some fish species could be partitioning habitat and garnering energy from more littoral or pelagic resources in the reservoir^42^. For instance, *D. cepedianum* and *X. texanus* might forage more in the littoral zone or shallower in the water column where food resources are less depleted^84–86^, which matches patterns observed in other food web and habitat use studies from Lake Powell^87,88^. The δ^13^C signature of *X. texanus* captured in the river downstream of the waterfall align more with that from the reservoir than the river upstream, which was not surprising since this species moves in the river for only a few weeks or months (M. Bogaard, unpublished data), spending most of their time in the reservoir^87^.

Many native fishes in the Colorado River Basin, as well as the nonnative species we assessed, are considered trophic generalists. All fishes, native and nonnative, appeared to be feeding on primary consumers and higher trophic levels (e.g., predatory macroinvertebrates, smaller fishes, zooplankton) rather than feeding directly on phytoplankton or benthic algae in the reservoir, assuming trophic fractionation of 3.4 ‰ in δ^15^N^45^. For instance, the δ^15^N of filter-feeding quagga mussels collected from the reservoir reach during our study had a δ^15^N of 6.3 ± 1.1 (mean ± SE) and much more variable δ^13^C (-33.1 ± 2.3). Previous research on isotopic niches of native fishes in the Colorado River basin have mainly used smaller-bodied individuals relative to those we used here (e.g.,^53^), limiting our ability to make direct comparisons of isotopic signatures. Whereas trophic position of fish tends to increase with size^89^; the random effect of fish length explained relatively little variation in our models. Nonetheless, our δ^15^N values for *P. lucius* (total length range: 222–526 mm), *I. punctatus*, and *C. latipinnis* are comparable to those reported previously from the San Juan River (range: 100–350 mm TL)^90^, although body sizes were not reported for the latter two species.

Niche breadth varied spatially among the three habitats for native and nonnative species, presumably as the major resource base shifted from allochthonous to autochthonous^26,82,91^. Somewhat surprisingly, on average, native fishes demonstrated consistent trophic niche breadth (based on SEAc) among the three habitats. All native fishes demonstrated substantially wider niches in the river upstream relative to nonnative fishes, and although the niche breadth of some nonnative species increased moving downstream towards the reservoir (i.e., *C. carpio* and *I. punctatus*), *X. texanus* maintained a relatively wide niche breadth across habitats. In all three habitats, niche breadth was generally influenced more by variation in δ^13^C among individuals within species (based on the ratio of SD of δ^13^C to SD of δ^15^N; most values > 1.4), potentially due to variation in basal resource (only measured in the reservoir) or habitat use. Only *C. carpio* in the river upstream and downstream and *I. punctatus* in the river downstream appeared to have niche breadth dually influenced by variation in δ^13^C and δ^15^N (ratio values = 0.8–1.1). Overall, species exhibited shifts towards more depleted carbon isotope sources moving from the river upstream (mean ± SD; − 22.5 ± 2.6) to the reservoir (− 27.5 ± 3.5), but the amount of overlap in isotopic niche space among species varied across habitats.

Human transformation of riverscapes has restructured fish assemblages with consequences for the conservation of native species. Artificial habitats, such as reservoirs and isolated river fragments, are now common^92^, and native and nonnative species co-occur in these habitats^93^, with nonnative species often thriving in more modified habitat^51,94,95^. In the Colorado River basin, native fish assemblages have endured habitat loss and degradation alongside the introduction and establishment of nonnative species, but recovery efforts are hindered by a full accounting of limiting factors, particularly the importance of biotic interactions. Although we observed greater niche overlap between native and nonnative fishes in habitats with a greater relative abundance of nonnative fishes (i.e., the river-reservoir inflow), it remains unclear whether this overlap has negative impacts on native populations. It is difficult to assess the importance of overlap between native fish and potential nonnative competitors because data on resource availability are lacking across the basin. This study adds to a growing body of research suggesting adult native fishes may be able to coexist with some nonnative fishes that also have an opportunistic feeding strategy. In addition, we demonstrate some native fishes might be able to successfully utilize highly modified river-reservoir inflow and reservoir habitat^51,95,96^, habitats similar to lotic-lentic habitats that have been present across their evolutionary history.

## Supplementary Information

Supplementary Information.
