# Supplementary material for: Trophic niches of native and nonnative fishes along a river-reservoir continuum
Source: Sci Rep. 2021 Jun 9;11:12140. doi: 10.1038/s41598-021-91730-1 (PMC8190098; doi:10.1038/s41598-021-91730-1)
Supplement: Supplementary file 1 — Supplementary Information. [file 41598_2021_91730_MOESM1_ESM.docx]

*Electronic Supplementary Material*

Trophic niches of native and nonnative fishes along a river-reservoir continuum

Casey A. Pennock^1,2^*, Zachary T. Ahrens^1,3^, Mark C. McKinstry^4^, Phaedra Budy^5,1^, Keith B. Gido^2^

^1^Department of Watershed Sciences and The Ecology Center, Utah State University, Logan, UT 84322

^2^Division of Biology, Kansas State University, Manhattan, KS 66502

^3^Utah Division of Wildlife Resources, Moab, UT 84532

^4^Upper Colorado Regional Office, U.S. Bureau of Reclamation, Salt Lake City, UT 84138

^5^U.S. Geological Survey, Utah Cooperative Fish and Wildlife Research Unit, Utah State University, Logan, UT 84322-5290

^*^corresponding author: [casey.pennock@usu.edu](mailto:casey.pennock@usu.edu)

Supplementary Table S1: Mean δ^13^C and δ^15^N, fish size (mm total length), and number of tissues analyzed (*n*) from fishes captured in the San Juan River and Lake Powell, Utah downstream of the Piute Farms Waterfall that were not included in the analyses because they were only captured in one of the habitats. Native species are denoted with an *.

| Habitat/Species | δ^13^C  (mean, SE) | δ^15^N  (mean, SE) | Total length  (mean ± SD) | *n* |
| --- | --- | --- | --- | --- |
| *River downstream* |  |  |  |  |
| Red Shiner (*Cyprinella lutrensis*) | -25.1 (0.4) | 9.9 (0.3) | 55 ± 22 | 20 |
| Striped Bass (*Morone saxatilis*) | -26.6 (0.7) | 15.8 (0.7) | 521 ± 33 | 7 |
| Walleye (*Sander vitreus*) | -25.3 (0.6) | 14.3 (0.4) | 484 ± 38 | 10 |
|  |  |  |  |  |
| *Reservoir* |  |  |  |  |
| Black Bullhead (*Ameiurus melas*) | -31.8 | 10.5 | 238 | 2 |
| Yellow Bullhead (*Ameiurus natalis*) | -27.4 (0.4) | 14.4 (0.3) | 258 ± 27 | 20 |
| Bluehead Sucker (*Catostomus discobolus*)* | -22.2 | 12.9 | 292 | 1 |

Supplementary Table S2: Months in which tissues were collected from fishes captured in the San Juan River and Lake Powell.

| Habitat/Species | Months of tissue collection |
| --- | --- |
| River upstream |  |
| Channel Catfish (*Ictalurus punctatus*) | 3,4,9 |
| Colorado Pikeminnow (*Ptychocheilus lucius*)* | 3,4,9 |
| Common Carp (*Cyprinus carpio*) | 4,9 |
| Flannelmouth Sucker (*Catostomus latipinnis*)* | 9 |
| Razorback Sucker (*Xyrauchen texanus*)* | 3,4 |
|  |  |
| River downstream |  |
| Channel Catfish (*I. punctatus*) | 3,6,9 |
| Colorado Pikeminnow (*P. lucius*) | 3,6,9 |
| Common Carp (*C. carpio*) | 3,9 |
| Flannelmouth Sucker (*C. latipinnis*)* | 3,9 |
| Gizzard Shad (*Dorosoma cepedianum*) | 3,9 |
| Razorback Sucker (*X. texanus*)* | 3,9 |
|  |  |
| Reservoir |  |
| Channel Catfish (*I. punctatus*) | 4,5 |
| Common Carp (*C. carpio*) | 4,5 |
| Flannelmouth Sucker (*C. latipinnis*)* | 4,5 |
| Gizzard Shad (*Dorosoma cepedianum*) | 4,5 |
| Razorback Sucker (*X. texanus*)* | 4,5 |


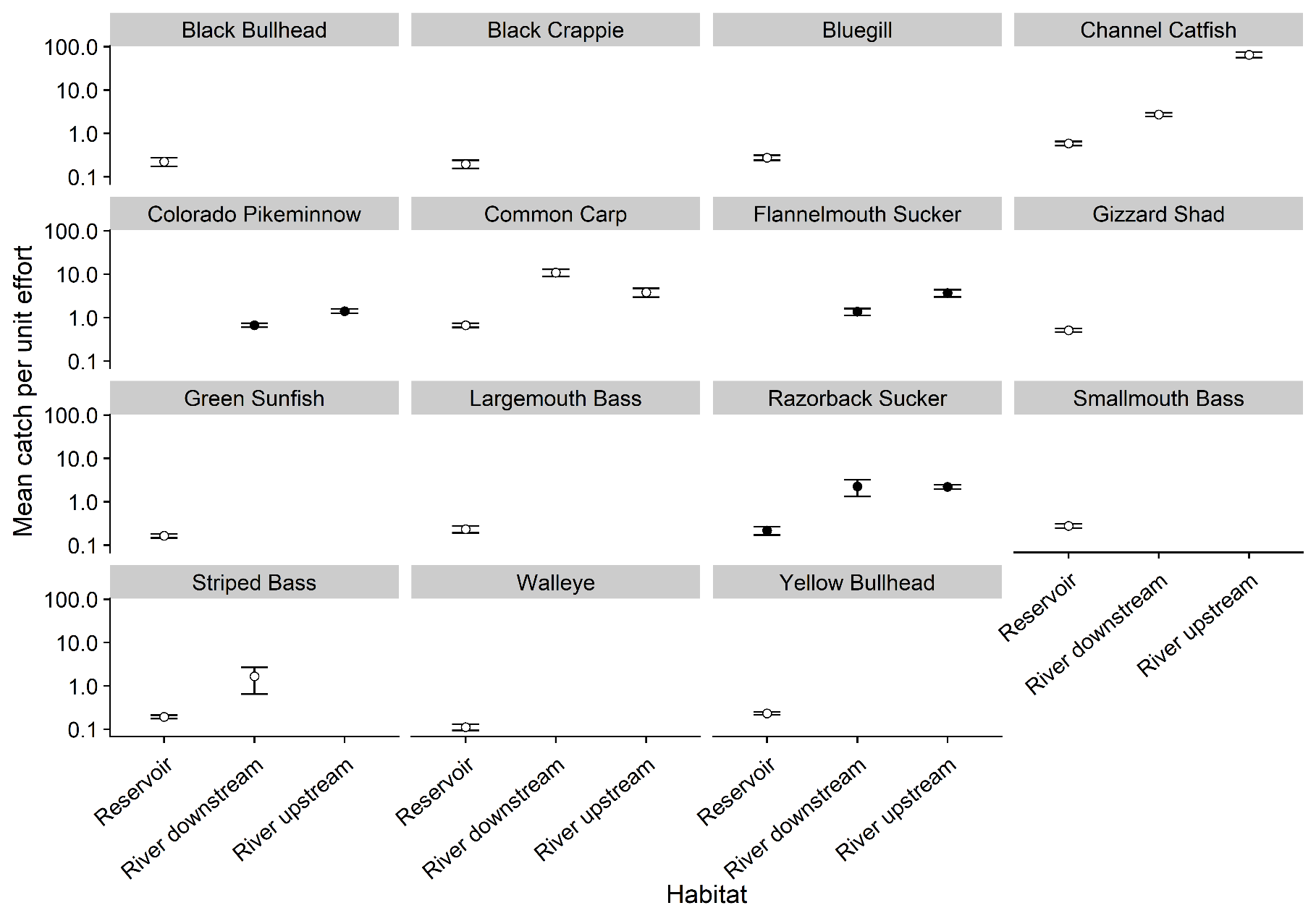


Supplementary Fig. S1: Catch per unit effort (fish h-1; mean ± SE) of species making up at least 1% of total fishes captured in each habitat. Native species are represented by black circles and nonnative species are white circles. Riverine habitats were sampled with raft electrofishing and the reservoir was sampled with trammel nets.

Supplementary Fig. S2: Regression of δ^13^C (‰) of quagga mussels versus river kilometer where mussels were collected along the San Juan River-Lake Powell inflow area from reservoir habitat (see Figure 1 in main manuscript). Mussels were collected within 1 m of the surface.
